# Impact of environmental cleaning on the colonization and infection rates of multidrug**-**resistant *Acinetobacter baumannii* in patients within the intensive care unit in a tertiary hospital

**DOI:** 10.1186/s13756-020-00870-y

**Published:** 2021-01-06

**Authors:** Yang Li, Hai Ge, Hui Zhou, Wanqing Zhou, Jie Zheng, Wei Chen, Xiaoli Cao

**Affiliations:** 1 Department of Nosocomial Infection Control, Nanjing Drum Tower Hospital, The Affiliated Hospital of Nanjing University Medical School, Nanjing, 210008 Jiangsu People’s Republic of China; 2 Department of Laboratory Medicine, Nanjing Drum Tower Hospital, The Affiliated Hospital of Nanjing University Medical School, Zhongshan Road 321, Gulou, Nanjing, Jiangsu Province People’s Republic of China; 3 Clinical Research Center, The Second Hospital of Nanjing, Nanjing University of Chinese Medicine, Zhongfu Road 1-1, Gulou, Nanjing, 210003 People’s Republic of China

**Keywords:** Healthcare-associated infection, Multidrug resistance, *Acinetobacter baumannii*, Fluorescence labeling, Environmental cleaning

## Abstract

**Objective:**

To continuously evaluate the effect of environmental cleaning and hand hygiene compliance on the colonization and infection rates of multidrug-resistant *Acinetobacter baumannii* (MDR-AB) in the patients within an intensive care unit (ICU).

**Methods:**

Environmental cleaning on the high-touch clinical surfaces (HTCS) within a comprehensive ICU was evaluated through monitoring fluorescent marks when the overall compliance with hand hygiene during 2013–2014 was monitored. Meanwhile, samples from the HTCS and inpatients were collected and sent for bacterial culture and identification. The drug susceptibility testing was further implemented to monitor the prevalence of MDR-AB. The genetic relatedness of MDR-AB collected either from the HTCS or inpatients was analyzed by pulsed field gel electrophoresis (PFGE) when an outbreak was doubted.

**Results:**

The overall compliance with hand hygiene remained relatively stable during 2013–2014. Under this circumstance, the clearance rate of fluorescence marks on the environmental surfaces within ICUs significantly increased from 21.9 to 85.7%, and accordingly the colonization and infection rates of MDR-AB decreased from 16.5 to 6.6‰ and from 7.4 to 2.8‰, respectively, from the beginning to the end of 2013. However, during 2014, because of frequent change and movement of environmental services staff, the clearance rate of fluorescence marks decreased below 50.0%, and the overall colonization and infection rates of MDR-AB correspondingly increased from 9.1 to 11.1‰ and from 1.5 to 3.9‰, respectively. PFGE displayed a high genetic relatedness between the MDR-AB strains analyzed, indicating a dissemination of MDR-AB during the surveillance period.

**Conclusion:**

For the easily disseminated MDR-AB within ICUs, the clearance rate of fluorescence labeling on HTCS is negatively correlated with the hospital infection rate of MDR-AB. Such an invisible fluorescence labelling is an effective and convenient method to continuously monitor cleanness of medical environment within hospitals.

## Introduction

Healthcare-associated infection (HAI) is a global problem for patients, especially those inpatient with immunocompromised or critically ill diseases, causing extended hospital stays, high costs, and high mortality [1]. Epidemiological studies showed that HAI is closely associated with microbial pathogens, such as methicillin-resistant *Staphylococcus aureus* (MRSA) [2, 3], vancomycin-resistant *Enterococci* (VRE) [3], and multidrug-resistant Gram-negative bacilli [4], which could be spread by the hospital environment surfaces [5]. In USA and Europe, MRSA and VRE are the main pathogens associated with HAIs within intensive care units (ICUs) [3, 6, 7]. In contrast, in China, the prevalence of multidrug-resistant *Acinetobacter baumannii* (MDR-AB) greatly exceeds both MRSA and VRE [8, 9], and the frequent expansion of MDR-AB poses tough challenges to control HAIs, especially those occurred in ICUs [10, 11]. *A. baumannii* possesses the super survivability on kinds of healthcare equipment surfaces, from 5 days to more than 5 months [12], which surely increases the chance of transmission of MDR-AB. In addition, such strain is difficult for prevention once it acquires resistance to the conventional detergents and alcohol disinfectants [13]. The worrying condition is, the current arsenal to target MDR-AB is almost exhausted [14]. Therefore, management of *A. baumannii* clusters in hospitals is very important to control multidrug-resistant infections. The targeted infection control measures of MDR-AB, including hand hygiene, environmental cleaning and subsequent measurement of cleanliness, are imperative to eradicate the nosocomial acquisition and further dissemination.

As an essential infection prevention strategy, environmental cleaning has significant benefit for healthcare units that have either high or low hand hygiene compliance levels, albeit hand hygiene remains a priority for infection control programs [15]. To date, it’s known that the patient care items and high-touch clinical surfaces (HTCS) within hospital wards are blamed for pathogen transmission [16]. HTCS refer to the guardrail, bedside table, injection pump button, monitor button, treatment vehicle and treatment table, basing on hand contact frequency and easy-to-contact area of patients, which was specifically recommended in 2002 by the Centers for Disease Control and Prevention (CDC) [16]. Thus, the HTCS are always the focus of intensive cleaning in high-risk areas, especially when MDR-AB is epidemic or endemic, and the cleaning and disinfection of HTCS are pivotal for prevention of outbreak of MDR pathogens.

Considering the fluorescent marker does not allow for direct assessment of the degree of disinfection, previous studies showed that an invisible fluorescent marker is a much better strategy to improve environment cleaning, by quantitatively assessing cleaning and disinfecting practices on MRSA and VRE [17, 18]. However, to our best knowledge, there is no report on this method used to evaluate impact of hospital environmental cleaning in China, especially for MDR-AB*.*

In this study, we utilized an invisible fluorescent marker, together with bacterial culture and identification, to evaluate the environmental cleaning on the HTCS within a comprehensive ICU of Nanjing Drum Tower Hospital. Concurrently, hand hygiene compliance was measured, the impact of environmental cleaning and hand hygiene compliance on the colonization and infection rates of MDR-AB in patients from 2013 to 2014 was explored. In addition, the relationship between colonization rates and infection rates was also analyzed.

## Materials and methods

### Study design

The study was conducted within Nanjing Drum Tower Hospital, a 3,325-bed general tertiary care and university-affiliated teaching hospital in Nanjing, Jiangsu province, China. Ethical approval was approved by the Ethics Committee of Nanjing Drum Tower hospital (Number: 2013–042).

**Fig. 1:**
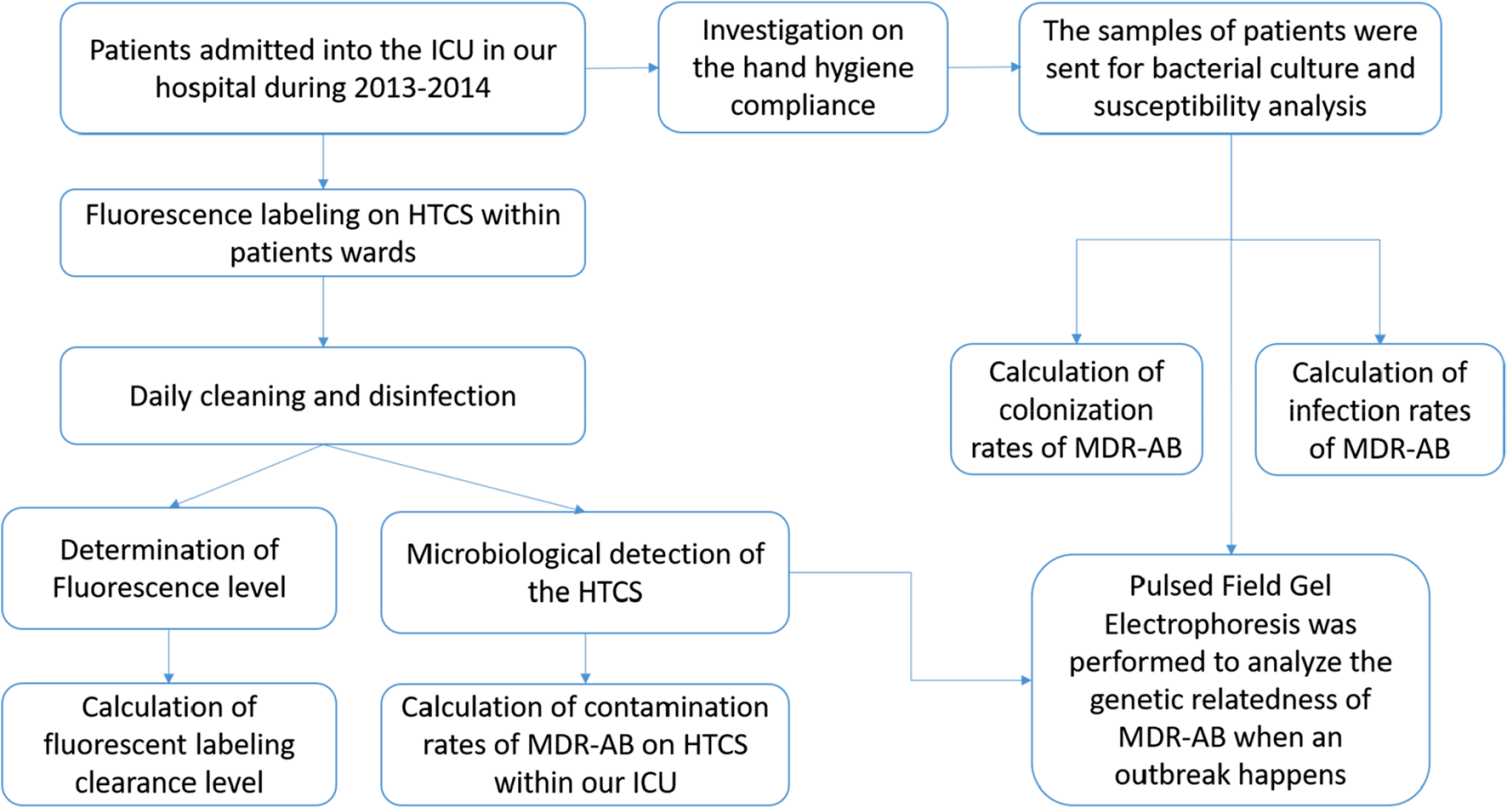
The study design of this study. ICU: Intensive Care Unit; MDR-AB: multidrug resistant *Acinetobacter baummannii*; HTCS, high-touch clinical surfaces

The study design was shown in Fig. 1. Briefly, the samples of patients hospitalized in a 27-bed ICU in our hospital were taken to monitor MDR-AB from 2013 to 2014. The HTCS were marked by fluorescence labeling and continuously monitored for clearance level of fluorescence labeling and for contamination rate of MDR-AB, which was resistant against at least one agent in three or more of tested antimicrobial categories [19].

### Investigation on the hand hygiene compliance

Before this research project, all healthcare personnel in our hospital were regularly required to perform hand hygiene according to the guidelines recommended by Centers for Disease Control and Prevention (CDC) [20]. Therefore, observational survey of compliance with hand hygiene were conducted without hand hygiene being mandatorily requested during 2013–2014. Hand hygiene opportunities were as follows: [1] before touching a patient, [2] before clean/aseptic procedures, [3] after body fluid exposure/risk, [4] after touching a patient, and [5] after touching patient surroundings according to recommended guidelines [21–23]. An alcohol-based hand rub or wash with soap and water should been used according to the special indications for hand hygiene [24]. Twenty-minute observations were conducted for 4–6 times every week at optional time periods of day or night throughout the week by a well-trained nurse who was as unobtrusive as possible, but was not hidden. The potential opportunities for hand hygiene and the actual number of episodes of handwashes or hand rubs were recorded, other related information was also noted on a standardized observation form of hand hygiene [25]. The Healthcare personnel did not know the schedule of observation periods, they will be reminded if bad/harmful practices were observed.

### Labeling of fluorescence marks and determination of the clearance level

The fluorescence marks were drawn by a fluorescent pen (RUHOF), which uses a special nontoxic target solution. When exposed to black light, the marks emit fluorescence brightly. Noteworthily, the fluorescence marks are inconspicuous, dry rapidly on surfaces, remain environmentally stable for several weeks, resist dry abrasion, but could be easily removed by minimal abrasion with moistened cloth [18, 26]. Fluorescence labeling was performed twice a day before cleaning (at 10 a.m. and 5 p.m. respectively), one mark every square centimeter. The targets were evaluated after two daily routine cleaning. Terminal cleaning was performed after inpatients were transferred from the ICUs. Clearance level of fluorescence labeling was calculated by comparing the number of fluorescence marks before and after cleaning. Environment cleanliness was divided into cleaning (labeling clearance rate > 80%) and contamination (labeling clearance rate < 80%).

### Monitoring contamination of MDR-AB on the HTCS

To monitor contamination of MDR-AB on the HTCS of the ICU before the daily cleaning, samples were accordingly taken for bacterial culture from each site of fluorescence labeling by a cotton swab moistened with saline, according to the Technical Specification for Disinfection of Hospital Disinfection Hygiene Standard issued by the Ministry of Health of China (http://www.biaozhun8.cn/biaozhun108760). Once *A. baumannii* was detected, antimicrobial susceptibility was further tested to detect MDR-AB.

### The colonization and infection rates of MDR-AB among inpatient in the ICU

Clinical samples including sputum, urine, blood, etc., from patients within the ICU during 2013–2014 were routinely taken and sent to the clinical microbiology laboratory for bacterial culture and susceptibility testing once infections were suspected. The diagnostic criteria for colonization and infection referred to the criteria issued by the US CDC in 2008 [27]. According to the international epidemiological quantitative statistical methods, the newly isolated multidrug-resistant bacteria per thousand bed days was adopted as the quantitative statistical standard, that is, the detection or infection density of multidrug-resistant bacteria in a specific time range (Number of newly isolated multidrug-resistant bacteria infected or colonized new patients in a period/number of bed days in a period).

### Bacterial identification and antimicrobial susceptibility testing

Strains isolated were identified by ATB32E or Vitek-2 technology (BioMerieux, France). The susceptibility was determined by Kirby-Bauer method. The tested antimicrobial agents were as follows: amikacin, ceftazidime, cefoperazone/sulbactam, imipenem, meropenem, piperacillin-tazobactam, cefepime, ticarcillin/clavulanate, ciprofloxacin, levofloxacin, sulfamethoxazole, minocycline and tigecycline. *Escherichia coli* American Type Culture Collection (ATCC) 25,922 and *Pseudomonas aeruginosa* ATCC27853 were used as the quality controls in parallel. The results were interpreted according to guidelines of Clinical Laboratory Standard Institute (CLSI) 2015[28]. However, the interpretation of tigecycline was referred to the guidelines of the current European Committee on Antimicrobial Susceptibility Testing (EUCAST) (www.eucast.org), cutoff MICs of ≤ 1 μg/ml and > 2 μg/ml were used for tigecycline as the susceptibility and resistance breakpoints, respectively.

### Pulsed field gel electrophoresis

When 14 MDR-AB isolates were detected simultaneously during Jan-Mar, 2013, the genetic relatedness among those MDR-AB strains collected from the patients and the HTCS during the same period were further analyzed through pulsed field gel electrophoresis (PFGE) according to the protocol [29]. Briefly. Fresh and pure bacterial cultures were embedded in agarose plugs and digested with proteinase K (20 mg/mL), followed by *Apa*I restriction endonuclease (TaKaRa, Dalian, Beijing, China). The standard strain *Salmonella enterica* serotype Braenderup H9812 digested with *Xba*I was used as a marker. The electrophoresis was performed in 0.5 × TBE buffer in a pulsed-field electrophoresis system (Chef Mapper; Bio-Rad Laboratories, Hercules, CA, USA), and the conditions were as follows: 14 °C, 6 V/cm, switch angle 120°, switch ramp 5–20 s for 19 h. BioNumerics software version 7.6 (Applied Maths, Sint-Martens-Latem, Belgium) was used to analyze the PFGE banding patterns. A cut off of 85% was used to judge the relatedness of strains analyzed based on the tree constructed by the unweighted pair group method of averages and a position tolerance of 1.5%.

### Statistical analysis

IBM SPSS Statistics 20.0 software was used to perform statistical analysis. To determine whether there are statistical outliers among the fluorescence label clearance rates, we performed multivariate linear regression analysis to check their Mahalanobis distance. Different marker numbers in the 8 quarters was tested for Normal distribution. The correlation between the removal level of fluorescence labeling and colonization rates of MDR-AB, and the relationship between the colonization rates and infection rates of MDR-AB were analyzed by the Spearman correlation analysis. P < 0.05 was taken as statistically significant.

## Results

### The compliance of hand hygiene in the comprehensive ICU

Totally, 676 opportunities for hand hygiene were recorded during 2013–2014 (Table 1). Clinician contributed a majority of 51.2% of all opportunities followed by nurse (36.0%) and environment service staff (12.9%). In general, adherence rates did vary by category of healthcare personnel. compliance of the clinicians was the best, whereas, the adherence of environment service staff was the worst. From 2013 to 2014, the compliance of clinicians increased from 68.6 to 76.6%, the adherence of nurses and environment service staff showed a fluctuating trend. However, the adherence rates kept relatively stable, and the average reached 61.8%.

**Table 1:** Hand hygiene compliance

Medical staff		Time period							
		1–3, 2013	4–6, 2013	7–9, 2013	10–12, 2013	1–3, 2014	4–6, 2014	7–9, 2014	10–12, 2014
Clinicians	Number of opportunities	51	34	43	43	42	48	38	47
	Number of executions	35	24	31	30	32	34	28	36
	Compliance rate (%)	68.6	70.6	72.1	69.8	76.2	70.8	73.7	76.6
Nurse	Number of opportunities	42	25	38	19	29	29	35	26
	Number of executions	24	14	18	9	19	16	20	18
	Compliance rate (%)	57.1	56.0	47.4	47.4	65.5	55.2	57.1	69.2
Environment service staff	Number of opportunities	9	7	18	3	8	15	12	15
	Number of executions	4	3	6	2	2	4	0	7
	Compliance rate (%)	44.4	42.9	33.3	66.7	25.0	26.7	0	46.7
Total	Number of opportunities	102	66	99	65	79	92	85	88
	Number of executions	63	41	55	41	53	54	48	61
	Compliance rate (%)	61.8	62.1	55.6	63.1	67.1	58.7	56.5	69.3

### Clearance of fluorescence labeling

At the initial stage, the clearance rate of fluorescence labeling was comparatively low, only 21.9% (Table 2). Through training and strengthening supervision of cleaning workers, the total clearance rate of fluorescence labeling was greatly improved and finally reached up to 85.7% at the last quarter of 2013. However, with the frequent change and mobility of environment service staff within our ICU during 2014, the average clearance rate sharply decreased to less than 50%, even though frequent straining and education were implemented.

**Table 2:** The number of fluorescent marks on high frequency clinical sites and clearance rates of fluorescence labeling

HTCS	The number of fluorescent markers and removal	Time period							
		1–3, 2013	4–6, 2013	7–9, 2013	10–12, 2013	1–3, 2014	4–6, 2014	7–9, 2014	10–12, 2014
Guardrail	Marker number	520	431	463	531	568	446	420	467
	Scavenging number	102	139	377	489	283	186	175	173
Bedside table	Marker number	116	124	112	127	108	91	74	85
	Scavenging number	23	89	100	115	75	47	44	31
Treatment vehicle	Marker number	132	97	144	105	87	96	111	93
	Scavenging number	54	73	124	87	39	39	57	55
Monitor button	Marker number	194	108	137	99	115	127	100	86
	Scavenging number	31	43	87	76	47	53	41	26
Injection pump button	Marker number	191	84	154	116	152	110	242	131
	Scavenging number	58	27	119	84	70	31	73	48
Treatment table	Marker number	46	18	33	29	19	15	41	62
	Scavenging number	1	7	28	12	5	6	17	21
total	Marker number	1199	862	1043	1007	1049	885	988	924
	Scavenging number	269	378	835	863	519	362	407	354
Clearance rate (%)		21.9	43.9	80	85.7	49.5	40.9	41.2	38.3

Statistical analysis was conducted for the different numbers of fluorescent marks among the 8 quarters fluctuating from 862 to 1199, which showed that all of them conformed to the normal distribution (p = 0.2)

### The contamination rate of MDR-AB on the HTCS within ICU

To monitor the contamination level of MDR-AB on the HTCS, samples were collected for bacterial culture and identification. The distribution of MDR-AB isolates on HTCS was displayed in Table 3. At the first quarter of 2013, 6 MDR-AB isolates were detected from HTCS, mainly on treatment vehicle and guardrail. With the increasing clearance rate of fluorescence labeling, the contamination rate of MDR-AB decreased remarkably. Thus, in the following 3 quarters, no MDR-AB isolates were found. However, with the drastic fluctuation of fluorescence clearance rate during 2014, the MDR-AB isolates were continuously detected from guardrail, treatment vehicle and treatment table.

**Table 3:** Distribution of multidrug-resistant *Acinetobacter baumannii* on HTCS

HTCS	The number of fluorescent markers and removal	Time period							
		1–3, 2013	4–6, 2013	7–9, 2013	10–12, 2013	1–3, 2014	4–6, 2014	7–9, 2014	10–12, 2014
Guardrail	Number of samples	10	10	10	8	8	8	8	8
	Number of positive samples	2	0	0	0	1	0	1	1
Bedside table	Number of samples	6	5	4	3	3	3	4	4
	Number of positive samples	0	0	0	0	0	0	0	0
Treatment vehicle	Number of samples	8	10	8	6	4	4	6	8
	Number of positive samples	3	0	0	0	0	0	1	1
Monitor button	Number of samples	3	2	3	3	4	4	3	3
	Number of positive samples	0	0	0	0	0	0	0	0
Injection pump button	Number of samples	3	2	3	3	4	4	3	3
	Number of positive samples	0	0	0	0	0	0	0	0
Treatment table	Number of samples	3	3	3	3	4	5	6	6
	Number of positive samples	1	0	0	0	0	1	1	0
total	Number of samples	33	32	31	26	27	28	30	32
	Number of positive samples	6	0	0	0	1	1	3	2
Positive rate (%)		18.2	0	0	0	3.7	3.6	6.7	6.3

### The colonization and infection rates of MDR-AB among inpatient within the ICU

As shown in Table 4, in 2013, the hospital colonization rate of MDR-AB per Bed Day changed in the wake of the clearance level of fluorescence labeling. Spearman correlation analysis found a significant association between them (*p* = 0.021). In addition, we found that the infection rates of MDR-AB changed along with the colonization rates of inpatient within the ICU. However, we did not find a significant correlation between the colonization rates and the infection rates of MDR-AB.

**Table 4:** The clearance rates of fluorescent marks, the colonization rates of multidrug-resistant *Acinetobacter baumannii* and the infection rates of multidrug-resistant *Acinetobacter baumannii* of the inpatients within our ICU

Time period	Fluorescent label clearance rates (%)	Bed days	The number of MDR-AB for colonization of inpatients	The number of MDR-AB for healthcare-associated infections	The daily colonization rates of MRD-AB per thousand bed (‰)	The hospital infection rates of MDR-AB per thousand bed days (‰)
1–3, 2013	21.9	1761	29	13	16.47	7.38
4–6, 2013	43.9	1845	33	3	17.89	1.63
7–9, 2013	80	1542	14	7	9.08	4.54
10–12, 2013	85.7	1775	10	5	5.63	2.82
1–3, 2014	49.5	1972	18	3	9.13	1.52
4–6, 2014	40.9	1824	30	12	16.45	6.58
7–9, 2014	41.2	1779	15	5	8.43	2.81
10–12, 2014	38.3	1810	20	7	11.05	3.87

Overall, the increased clearance rate of fluorescence marks leads to decreased contamination rate of MDR-AB on HTCS. The colonization rate and infection rate of MDR-AB in inpatient also decreased correspondingly. Statistical analysis showed that the correlation equation between clear rates of fluorescence labeling HTCS and hospital infection rate per thousand bed days was $y=-0.0353x+5.666$, which indicated that the latter decreased along with the former’s increase $(\mathrm{Table }4)$. It’s worthy to mention that the clearance rate of January to March in 2013 (21.9%) was not statistical outlier, since its Mahalanobis distance was 5.31, which was less than the threshold value of chi-square test (16.74) during multivariate linear regression analysis.

### The genetic relatedness of the MDR-AB strains during the infection outbreak

**Fig. 2:**
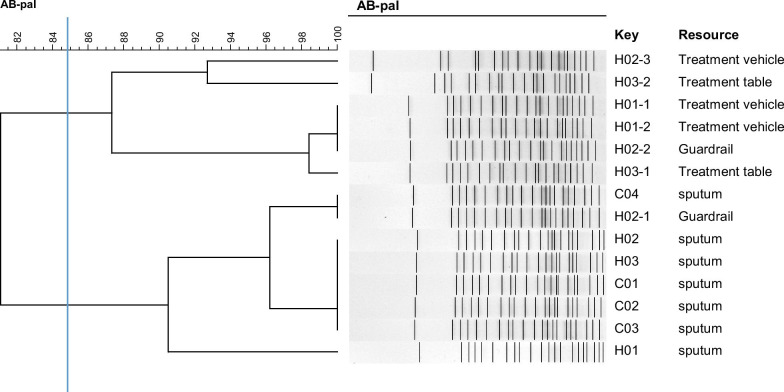
The genetic relatedness of the multidrug resistant *Acinetobacter baummannii* from different resources within the ICU. Dendrogram based on PFGE profiles of 12 multidrug-resistant *Acinetobacter baumannii* isolated from inpatients and environment surfaces within the ICU. The dendrogram was produced by the UPGMA algorithm based on the Dice similarity coefficient. H01, H02 and H03 were associated with hospital acquired infections; C01, C02, C03 and C04 were associated with community acquired infections; H01-1, H01-2 were isolated from the HTCS of H01 infected patient; H02-1, H02-2, H02-3 from the HTCS of H02 infected patient; H03-1 and H03-2 from the HTCS of H03 infected patient

From Jan to Mar in 2013, 7 MDR-AB strains were isolated from sputum samples of 7 inpatients. Among them, 3 strains were associated with HAIs (The positive sputum culture was drawn > 2 days after admission) [30], 4 strains with community infections (the positive sputum culture was drawn < 2 days after admission). At the same time, 7 MDR-AB strains were isolated from the HTCS of the 3 HAI patients (H01-H03). Among them, two were isolated from treatment vehicle of Patient H01, two from guardrail and one from the treatment vehicle of Patient H02, and the last two from treatment table of Patient H03. According to the cutoff of 85.0%, all the MDR-AB displayed a genetic relatedness, indicating the existence of an epidemic clone (Fig. 2).

## Discussion

In this study, we utilized a fluorescence labeling method to systematically evaluate the cleanliness of environmental surfaces within a comprehensive ICU in Nanjing Drum Tower Hospital, a large tertiary hospital of Nanjing, southeast China. Furthermore, the effect of environmental cleaning and hand hygiene adherence on the colonization and infection rates of MDR-AB in patients was also investigated. We found that the cleanliness of environmental surfaces could be reflected by clearance rate of fluorescence labeling on HTCS, under the conditions of keeping environment serve staff stable and relatively stable hand hygiene compliance. In addition, the clearance rate of fluorescence labeling could be greatly improved by training and strengthening supervision of environmental services staff. The increase of clearance rate of fluorescence labeling on HTCS was associated with the reduction of the hospital infection rate of MDR-AB.

Hand hygiene and environmental cleaning have previously been demonstrated to be two pillars of infection prevention in the control of hospital-acquired infection [15]. Comparing with the progressively improved compliance ( from 48.0% to 66.0%) in a teaching hospital in Switzerland during a 3-year survey period [31], and the low adherence to hand hygiene of clinicians (30.7%) at Queen Elizabeth Central Hospital in Malawi [25], the overall compliance with hand hygiene in our study was relatively stable and high (61.8%), albeit there were higher rates of compliance in clinicians when compared to nurse and environmental services staff. Notably, we found a continuously increasing compliance of hand hygiene of clinicians, which suggested that more and more clinicians in our ICU have realized the importance of the hand hygiene in the control of nosocomial pathogen. Considering the relatively stable hand hygiene adherence, more attention was therefore payed to analyze the effect of environment cleaning produced by the removal of fluorescence labeling.

Compared with the 44.0% clearance rate of black-light marks at baseline on surfaces in ICUs in the United Kingdom [18], the quite low removal rate (21.9%) of fluorescence labeling in our study corresponded to a bad environmental cleaning, which mean that more than 50.0% of the HTCS that should be wiped were actually not cleaned, indicating that cleaning of environmental surfaces should be strengthened. Fortunately, when the data were fed back to the management department of environmental services staff every quarter, more training aiming at improvement scheme of environmental cleaning would be repeatedly performed immediately, and stricter supervision was also implemented. Thus, the clearance rate of fluorescence labeling greatly increased up to 85.7% in the last quarter of 2013, a little lower than the 94% removal rate of fluorescent marker in United kingdom [32], suggesting that education of environmental services staff, and feedback using fluorescence labeling monitoring system could greatly improve the thoroughness of environment cleaning. However, because of the frequent change and mobility of environmental services staff in 2014, the environment cleaning dramatically fell, which was reflected by constantly decreased clearance rate of fluorescence labeling, even though more training and stricter supervision was still implemented. Accordingly, our study suggested that keeping the employment of environmental services staff stable within ICUs was especially important. Altogether, based on the fact that there are always much more patients and fewer beds in the large Third-Class A General Hospitals in China, it is difficult to achieve isolation measures strictly, such as single-room for each patient or the same-room placement for the same multidrug-resistant bacterial infections or colonizers. Thus, hand hygiene in combination with the formulated practical measures and training for all kinds of medical personnel to strengthen environmental cleaning as well as keeping the stability of environment service staff are imperative to prevent transmission of drug-resistant bacteria. Fluorescence labeling is an economical and effective method to rapidly and effectively evaluate the environmental clearance within ICUs.

Our further analysis found that there was a negative correlation between the clearance rate of fluorescence labeling on HTCS and the hospital infection rate per thousand bed days on the whole, which indicated that higher clearance rate of fluorescence labeling could reduce hospital infection rate of MDR-AB within ICUs. Moreover, few MDR-AB colonization in inpatients corresponded to the gradually increased clearance rate of fluorescence labeling on HTCS during 2013, indicating that good environment cleaning on HTCS could result in decreases in patient colonization.

Furthermore, the close genetic relationship of the MDR-AB isolated from the environmental surfaces and specimens of inpatients suggested the existence of an epidemic MDR-AB clone, which could rapidly spread within ICUs once the environmental cleaning was not enough, further emphasizing the importance of the sanitation of hospital environment. Thus, strengthening the cleanness and disinfection of the HCTS and other infection prevention and control measures is an effective way to prevent and control the dissemination of MDR-AB within hospitals.

There are several limitations to this study. First, our study focused only on daily cleaning when the room was occupied not terminal cleaning after patient discharge. Second, we just cultured only a fraction of marked surfaces because of financial constraints. Third, anal swabs were not taken for the surveillance for MDR-AB colonization.

## Conclusion

Epidemic MDR-AB is a main pathogen easily colonizing on the HCTS within ICUs. With the fluorescence labeling method, the environmental cleanliness could be effectively reflected and educational intervention could also be objectively assessed. Furthermore, our study emphasized the importance of monitoring environment clearance and keeping stability of environment serve staff under the condition of relatively stable hand hygiene compliance.

## Data Availability

The datasets used and/or analyzed during the current study are available from the corresponding author on reasonable request.
