# Correction to: Impact of environmental cleaning on the colonization and infection rates of multidrug-resistant *Acinetobacter baumannii* in patients within the intensive care unit in a tertiary hospital

**DOI:** 10.1186/s13756-021-00904-z

**Published:** 2021-03-05

**Authors:** Yang Li, Hai Ge, Hui Zhou, Wanqing Zhou, Jie Zheng, Wei Chen, Xiaoli Cao

**Affiliations:** 1 Department of Nosocomial Infection Control, Nanjing Drum Tower Hospital, The Affiliated Hospital of Nanjing University Medical School, Nanjing, 210008 Jiangsu People’s Republic of China; 2 Department of Laboratory Medicine, Nanjing Drum Tower Hospital, The Affiliated Hospital of Nanjing University Medical School, Zhongshan Road 321, Gulou, Nanjing, Jiangsu Province People’s Republic of China; 3 Clinical Research Center, The Second Hospital of Nanjing, Nanjing University of Chinese Medicine, Zhongfu Road 1-1, Gulou, Nanjing, 210003 People’s Republic of China

## Correction to: Antimicrob Resist Infect Control (2021) 10:4 10.1186/s13756-020-00870-y

The original article mistakenly omits co-corresponding authorship status for co-author, Wei Chen.

Both Wei Chen and Xiaoli Cao should be considered corresponding authors, and Wei Chen’s correspondence details can be viewed in this Correction article accordingly.
